# A systematic review on the effectiveness of herbal interventions for the treatment of male infertility

**DOI:** 10.3389/fphys.2022.930676

**Published:** 2022-11-04

**Authors:** Muhammad Nabeel Shahid, Hassaan Shahzad Afzal, Bareerah Farooq, Muhammad Rehan Yousaf, Muhammad Rauf Ijaz, Talha Ali Shafqat, Tahir Mehmood Khan, Chin Fen Neoh, Qi Ying Lean, Allah Bukhsh, Mahmathi Karuppannan

**Affiliations:** ^1^ Department of Pharmacy Practice, Faculty of Pharmacy, Universiti Teknologi MARA (UiTM) Cawangan Selangor, Puncak Alam Campus, Shah Alam, Malaysia; ^2^ Department of Pharmacy Practice, Institute of Pharmaceutical Sciences, University of Veterinary and Animal Sciences, Lahore, Punjab, Pakistan; ^3^ School of Pharmacy, Monash University, Subang Jaya, Malaysia; ^4^ Vector-Borne Diseases Research Group (VERDI), Pharmaceutical and Life Sciences CoRe, Universiti Teknologi MARA (UiTM), Shah Alam, Malaysia; ^5^ Faculty of Pharmacy, Universiti Teknologi MARA (UiTM), Cawangan Pulau Pinang, Kampus Bertam, Shah Alam, Malaysia; ^6^ Monash University Malaysia, Subang Jaya, Malaysia

**Keywords:** male infertility, herbal interventions, systematic review, semen parameters, Hochu-ekki-to, *W. somnifera*, complementary and alternative medicine

## Abstract

**Background:** Male infertility is an emerging health issue in the world today. Surgical interventions for the treatment of male infertility are available but are quite expensive. Herbal interventions pose a popular alternative for the treatment of infertility. However, much has to be learned regarding their safety and efficacy.

**Objective:** The aim of the study was to investigate the efficacy of herbal interventions in male infertility and also assess the possibility of these interventions as complementary and alternative medicine (CAM) in the future.

**Method:** From inception until 16 December 2021, all articles emphasizing the efficacy of herbal interventions in the treatment of male infertility are included in this review. Seven databases are searched. The literature obtained is screened and extracted. Semen parameters, hormonal concentration, and conception are the outcomes of interest.

**Results:** A total of 19 articles were included in this review. Herbal interventions might improve semen parameters in males with infertility. Among all the interventions, Hochu-ekki-to and *W. somnifera* have shown the most promising results and should be studied further in a larger sample size.

**Conclusion:** This systematic review has demonstrated the efficacy of herbal interventions, especially Hochu-ekki-to and *W. somnifera*, in treating male infertility.

## Introduction

Infertility is defined as failure to achieve clinical conception despite unprotected intercourse for 1 year (Rowe et al., 2000). According to the World Health Organization (WHO), 12 months is the lowest reference limit for time to pregnancy (TTP) (Makar and Toth, 2002). According to surveys, a total of 48.3 million (15%) couples worldwide are affected by any kind of infertility, and higher infertility rates are observed in Central Europe and Africa (Kuoti Al-Rekabi et al., 2019). In Pakistan, the prevalence of infertility is approximately 22% (Sami and Saeed Ali, 2012). Males contribute to 20–70% of all infertile cases in Pakistan (Agarwal et al., 2015), while in the United States, (US) 9% of males aged 15–44 years are affected by infertility (Esteves et al., 2012). Lack of knowledge about the factors affecting fertility in males ultimately causes some males to engage in various unethical activities that affect their biological life and their ability to produce sufficient healthy sperms for successful pregnancy in a woman (Chachamovich et al., 2010).

The etiology of male infertility is multifactorial. A total of 60% of all male infertility cases is caused by sperm dysfunction (Wright et al., 2014). Poor sperm quality and reduced sperm count are the major factors in male infertility. Sperm quality can be assessed by three primary endpoints, that is, sperm concentration, morphology, and motility (Buhling et al., 2019). Male infertility may be caused by urogenital infections, chromosomal abnormalities, urogenital carcinoma (Wang et al., 2020a), alteration in genital hormones (Dutta et al., 2019), and unhealthy lifestyle (Daumler et al., 2016). In 30–80% of cases, sperm damage is postulated to have resulted from oxidative stress caused by reactive oxidative species (ROS). This oxidative stress damages the DNA of sperms which ultimately decreases sperm motility, damages the membrane of the acrosome, and decreases the ability of the sperm to fertilize the oocyte (Barati et al., 2020).

Various treatment modalities including pharmacological and herbal interventions, laboratory methods, and surgical interventions are available to treat male infertility (Sinclair, 2000). Among the interventions, many couples opt for complementary and alternative medicines (CAM) due to their availability, affordability, and accessibility (Smith et al., 2010). The herbal products are believed to have several nutritional values and biological effects in treating male infertility (Abdi et al., 2017). Several plants contain flavonoids and phenolic compounds which are potent antioxidants and are highly effective in improving the quality of sperms (Rekka et al., 1996). Recently, the European Association of Urology has acknowledged the use of complementary herbal medicines to treat male infertility (Nejatbakhsh et al., 2016). However, the World Health Organization (WHO) still has some concerns regarding the insufficient scientific knowledge on the compounds of the medicinal plants. Thus, it is necessary to identify the natural active biological compounds in plants that have specified positive effects on male infertility (Marbeen et al., 2005; Roozbeh et al., 2021). Following identification, the said compounds can then be extracted, purified, and formulated to be used in the future.

Male infertility is a prevalent issue in Pakistan, and the use of herbal interventions is also prevalent in the country for various diseases. Many people in Pakistan tend to lean toward herbal medicines rather than allopathic treatments. Moreover, no study has been conducted to evaluate the efficacy of herbal interventions in the treatment of male infertility. This systematic review is aimed to summarize the results of all the accessible studies that fulfill the inclusion criteria to determine the efficacy parameters of herbal interventions in male infertility.

## Methodology

### Literature searching techniques

Seven electronic databases (PubMed, Scopus, Cochrane Library, Embase, EBSCOhost, Ovid, and Google Scholar) were searched for data sources and strategies from inception until 31 December 2021. Medical Subject Headings [MeSH] and text terms were included for search terms in this review. The strategic search terms are “herbal medicine” or “herbal” or “herbalism” or “herbals” or “homeopathy” AND “infertility” or “subfertility” or “subfertile” or “azoospermia” or “oligospermia” or “oligozoospermia” or “oligoasthenoteratozoospermia” or “genital disease” or “genitalia” or “genital” or “low sperm count” or “semen.” Details of search strategies used for each database are provided in the supplementary files.

### Inclusion and exclusion criteria

The PICOT framework was adopted to define the inclusion criteria (Table 1). The inclusion criteria are as follows.

**TABLE 1:** PICOT table of included studies.

Category	Description
**Population**	Infertile adult males
**Intervention**	Any herbal intervention approved according to guidelines and mentioned in the studies that meet the inclusion criteria of this study
**Control**	Any placebo or comparator eligible for inclusion in this study
**Outcome**	Sperm concentration, sperm motility, sperm morphology, total serum FSH, total serum testosterone, and conception
**Time**	Inception to December 2021

Studies that were included comprise (Rowe et al., 2000) randomized control trials or cluster-randomized controlled trials; (Makar and Toth, 2002) different herbal interventions (including homeopathic) evaluating the efficacy of men infertility issues (Kuoti Al-Rekabi et al., 2019) directed at male patients (≥18 years) with infertility only; (Sami and Saeed Ali, 2012) experimental single-case studies; (Agarwal et al., 2015) an original study published in a peer-reviewed journal; and (Esteves et al., 2012) article in the English language. Cross-sectional studies, *in vitro* studies, study protocols, expert opinions, case reports/series, editorials, abstracts from conferences, review articles, and studies involving animals were excluded.

### Study selection

Two reviewers MRY and TAS screened titles and abstracts extracted from various databases using the well-defined selection criteria. Appropriate articles were then screened individually by the reviewers MNS and AB to access their inclusion eligibility. Resolution of disagreement was primarily through discussion.

### Data extraction and qualitative synthesis

Data extraction was performed individually for all the selected articles by HSA, BF, and MRI. The extracted data were then reviewed by MNS and AB for proper extraction. Details about publication year, authors, design of the study, country, sample size, age of the patients, interventions, and outcomes of the study were extracted from each included study. The outcomes were summarized in the form of the changes in parameters from baseline up to the end of intervention for the patients of both the intervention and control (if any) groups. Any disagreement was resolved by a third reviewer if necessary.

### Risk of bias assessment

MNS and AB evaluated the risk of bias (ROB) of the included studies using the Cochrane ROB tool (Higgins et al., 2011). For RCTs, each ROB item was ranked as “low risk” if it was suspected that a bias would seriously alter the result, “unclear” if it was expected that a bias would raise some uncertainty about the results, or “high risk” if it was prospective that a bias would completely alter the result. A discussion was used to resolve disagreements among reviewers.

### Data analysis

Data analysis for the risk of bias was executed using Microsoft Excel 2010 and Review Manager 5.3.

## Results

**FIGURE 1:**
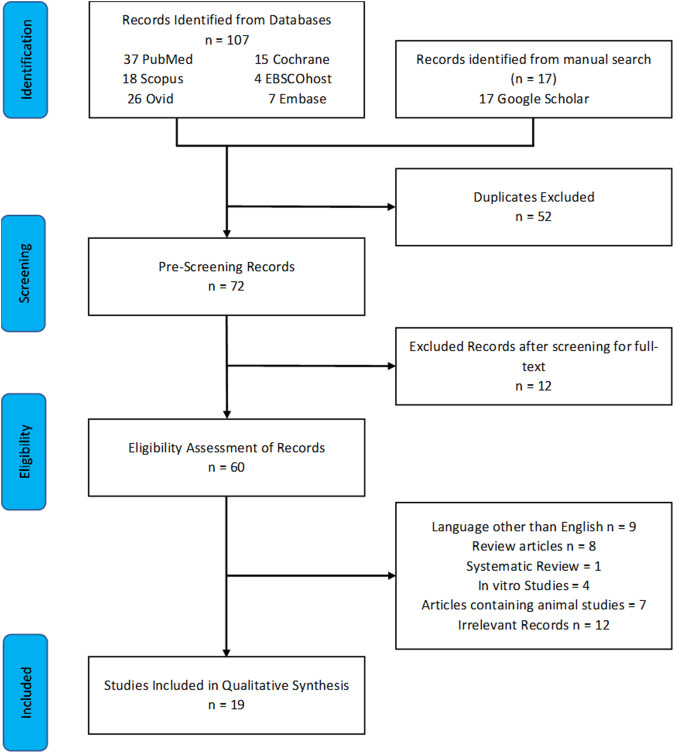
PRISMA flow chart.

A total of 124 articles were obtained, of which 107 were extracted from the databases mentioned and 17 were corkscrewed from Google Scholar. After the removal of 52 duplicate articles, 72 articles remained. Screening of the abstracts and titles of articles along with assessing the availability and relevancy of the full texts led to the removal of 12 articles. The remaining 60 articles were evaluated for eligibility based on the selection criteria, and 41 articles were excluded. Qualitative synthesis was performed for 19 studies. The details of these studies are depicted in the PRISMA flow diagram (Figure 1).

Out of the 19 selected articles, 6 were blinded RCTs (one triple-blind and five double-blind) and 13 were non-blinded RCTs. Eight studies were from Iran (Safarinejad et al., 2011; Khoradmehr et al., 2014; Kolahdooz et al., 2014; Rasekh et al., 2015; Ouladsahebmadarek et al., 2016; Nasimi Doost Azgomi et al., 2018; Kolangi et al., 2019; Aghajani et al., 2021), three in China (Yang et al., 2001; Peng et al., 2014; Wang et al., 2020a), four in Japan (Ishikawa et al., 1992; Furuya et al., 2004; Akashi et al., 2008; Yu et al., 2017), three in India (Rege et al., 1997a; Ahmad et al., 2010; Mahajan et al., 2012), and one in South Korea (Park et al., 2016).

### Characteristics of participants

The inclusion criteria were met by 19 studies which comprised 1,398 participants. All of the participants were proven infertile according to the WHO criteria of infertility. Their sperm count was lower than 20 million per milliliter. The sample size of the chosen studies ranged from n = 20 to n = 260 (Table 2).

**TABLE 2:** Characteristics of studies using herbal intervention to treat male infertility (*n* = 19).

S.No.	Author (year)	Study design/type	Place of study	Mean age of patients (year)	Type of male infertility	Patients (n)							Daily dosage regimen	Duration of intervention	Target parameter	Effects on infertility	Adverse effects
						Total	Intervention			Placebo	Control	Drop out					
							1	2	3								
1	Aghajani et al. (2021)	RCT	Iran	35.57 ± 5.7 (carob group) 34.40 ± 5.9 (vitamin E group)	Oligozoospermia, asthenospermia, and teratospermia	60	30	30	—	—	—	10	Intervention 1: 7.5 ml carob syrup BID. Intervention 2: 100 mg vitamin E BID	3 months	Sperm count	Improved in both groups. Greater improvement in the carob group	Not reported
															Sperm morphology		
															Sperm motility		
															Testosterone		
														Conception		
2	Wang et al. (2020b)	RCT	China	32.0 ± 3.1 (control group) 31.2 ± 4.4 (treatment group)	Idiopathic asthenozoospermia	66	31	—	—	—	30	5	Control: 1 g levocarnitine BID. Intervention: 150 ml Qixiong Zhongzi decoction BID	12 weeks	Sperm motility	Increased	Cold, allergy, headache, hypomania, nausea, and increased appetite
															Semen volume	No change	
															Sperm density	No change	
															Conception	No change	
															Sperm motility	Increased	
															Semen volume	No change	
															Sperm morphology	No change	
															Total motile sperm count	Increased	
3	Kolangi et al. (2019)	Double-blind RCT	Iran	33.8 ± 5.4	Idiopathic male infertility	76	31	—	—	29	—	16	Placebo: 100 mg TDS. Sucrose intervention: 100 mg TDS A. *officinarum* rhizome	3 months	Sperm count	Increased	Cold, allergy, headache, hypomania, nausea, and increased appetite
															Sperm volume	No change	
															Sperm motility	No change	
4	Nasimi et al. (2018)	Triple-blind RCT	Iran	32.48 ± 5.49 (WS)	Idiopathic male infertility	100	46	45	—	—	—	9	Intervention 1: 5 g/day WS roots. Intervention 2: 800 mg/day PTX + placebo	90 days	Sperm count	Increased	Not reported
															Sperm motility	Increased	
															Sperm morphology	Improved	
															Semen volume	No effect	
															Conception	Successful	
5	Yu et al. (2017)	RCT	Japan	36.9	Poor sperm concentration and motility	54	17	15	—	—	12	10	Intervention 1: 1 can tomato juice (30 mg lycopene) OD. Intervention 2: 1 antioxidant capsule (600 mg vitamin C, 200 mg vitamin E, and 300 mg glutathione) OD	12 weeks	Sperm motility	Improvement in the tomato juice group. No improvement in the antioxidant group	Not reported
															Sperm concentration		
															Semen volume		
6	Ouladsahebmadarek et al. (2016)	Experimental (single-case study)	Iran	34.28 ± 8.99	Oligospermia	40	39	—	—	—	—	1	700 mg capsule OD containing *A. cepa, Z. officinale, O. basilicum, C. verum, C. sinensis* peel, *C. lanatus*, and *D. carota* seeds	6 months	Sperm count	Increased	Not reported
															Sperm motility	Increased	
															Sperm morphology	Improved	
															Conception	Successful	
7	Park et al. (2016)	Double-blind RCT	South Korea	25–45 years	Varicocele-induced infertility	80	38	—	—	39	—	3	**Intervention:** 3 500 mg red ginseng capsules daily	12 weeks	Sperm concentration	Increased	Cold, allergy, headache, hypomania, nausea, and increased appetite
															Sperm motility	Increased	
															Sperm morphology	Increased	
															Sperm vitality	Increased	
															Testosterone	No change	
8	Rasekh et al. (2015)	Experimental (single-case study)	Iran	31.20 ± 5.15	Idiopathic infertility	40	40	—	—	—	—	—	**Intervention:** 120 mg/kg date palm pollen powder capsule QOD	2 months	Sperm morphology	Increased	Not reported
															Semen volume	No change	
															Sperm count	Increased	
															Sperm motility	Increased	
9	Khoradmehr et al. (2014)	Experimental (single-case study)	Iran	32.22 ± 5.2 (treatment group) 33.79 ± 3.7 (control group)	Oligozoospermia, asthenozoospermia, teratozoospermia, and oligoasthenoteratozoospermia	62	32	—	—	30	—	—	**Intervention:** TOPALAF 3 times a week	3 months	Sperm motility	Increased	Not reported
															Sperm morphology	No change	
															Sperm count	Increased	
															Sperm volume	Increased	
															Conception	Successful	
10	Kolahdooz et al. (2014)	Double-blind RCT	Iran	N/A	Idiopathic male infertility	80	34	—	—	34	—	12	Placebo: 2.5 ml of liquid paraffin BD. Intervention: 2.5 ml of *N sativa* oil BD	2 months	Sperm count	Increased	Not reported
															Sperm motility	Increased	
															Sperm morphology	Increased	
															Semen volume	Increased	
11	Peng et al. (2014)	RCT	China	26.3 (treatment group) 25.6 (control Group)	Semen non-liquefaction male infertility	62	31	—	—	—	31	—	Control: comprehensive therapy. Intervention: comprehensive therapy + Gushenyutai plaster	8 weeks	Conception	Successful and increased	Not reported
															Sperm density	Successful and increased	
12	Mahajan et al. (2012)	Experimental (single-case study)	India	27–45	Oligozoospermia	20	10	—	—	—	10	—	250 mg/kg herbal composition of *M. pruriens*, *C. borivilianum*, and *E. campestris* BID	90 days	Sperm density	Increased	Not reported
															Sperm motility	Increased	
															Serum FSH, LH, and testosterone	Increased	
13	Safarinejad et al. (2011)	Double-blind RCT	Iran	28.6 ± 5.4	Idiopathic oligoasthenoteratozoospermia	260	114	—	—	116	—	30	Placebo: 30 mg starch BD. Intervention 1: saffron 30 mg BD	26 weeks	Hormones, semen volume, sperm count, conc., motility, and morphology	No significant improvement in the treatment group	Cold, allergy, headache, hypomania, nausea, and increased appetite
14	Ahmad et al. (2010)	Experimental (single-case study)	India	25–40	Unexplained infertility, oligozoospermia, and asthenozoospermia	150	75	—	—	—	75	—	5 g/day *W*. *somnifera* root powder with milk	3 months	Sperm concentration	Increased	Not reported
															Sperm motility	Increased	
															Semen volume	Increased	
															Lipid peroxides	Decreased	
															Testosterone	Increased	
15	Akashi et al. (2008)	Experimental study	Japan	N/A	Idiopathic oligozoospermia and asthenozoospermia	20	20	—	—	—	—	—	7.5 g/day Hochu-ekki-to	3 months	Sperm motility	Increased	Not reported
															Seminal RANTES	Decreased	
															TNF-α & IL-6	Decreased	
16	Furuya et al. (2004)	Experimental study	Japan	N/A	Idiopathic oligozoospermia and asthenozoospermia	22	22	—	—	—	—	—	7.5 g/day Hochu-ekki-to	3 months	Sperm concentration	Increased	Not reported
															Sperm motility	Increased	
															Seminal sFas	No change	
															IL-6 & IL-8	No change	
															Quality sperm	Improved	
															Motility sperm	Increased	
															Vitality	Increased	
															Sperm motility	Increased	
															Sperm morphology	Increased	
															Sperm vitality	No change	
															Semen volume	No change	
17	Yang et al. (2001)	Experimental (single-case study)	China	N/A	Spermatopathy	87	87	—	—	—	—	—	**Intervention:** Sheng Jing Zhong Zi Tang	N/A	Conception	Improved	Not reported
															Semen quality	Improved	
															Testosterone	Increased	
															FSH and LH	Increased	
18	Rege et al. (1997b)	Double-blind RCT	India	N/A	Oligospermia and azoospermia	40 (phase 1)	20	—	—	20 (phase 1)	—	—	1 cap BD Y-virilin	6 months	Sperm count	Increased	Not reported
															Sperm motility	No change	
						12 (phase 2)	6			6 (phase 2)					Serum FSH	No change	
															Conception	Successful	
19	Ishikawa et al. (1992)	Experimental (single-case study)	Japan	32.7	Unexplained male infertility, oligozoospermia, asthenozoospermia, and oligoasthenoteratozoospermia	67	63	—	—	—	—	4	7.5 g/day Hochu-ekki-to	3 months	Sperm concentration	Increased	Not reported
															Sperm motility	Increased	
															Serum FSH	No change	
															Sperm count	Increased	
															Sperm activity	Increased	

RCT, randomized control trial; BID, bis in die; BD, bis in die; TID, Tes die sumendus; WS, *Withania somnifera*; PTX, pentoxifylline; OD, omni in die; QOD, every other day; ZYD, Zhuanyindan; FSH, follicle-stimulating hormone; LH, luteinizing hormone; IL-6, interleukin-6; IL-8, interleukin-8; sFas, soluble fibroblast-associated; TNF, tumor necrosis factor-alpha.

### Risk of bias

**FIGURE 2:**
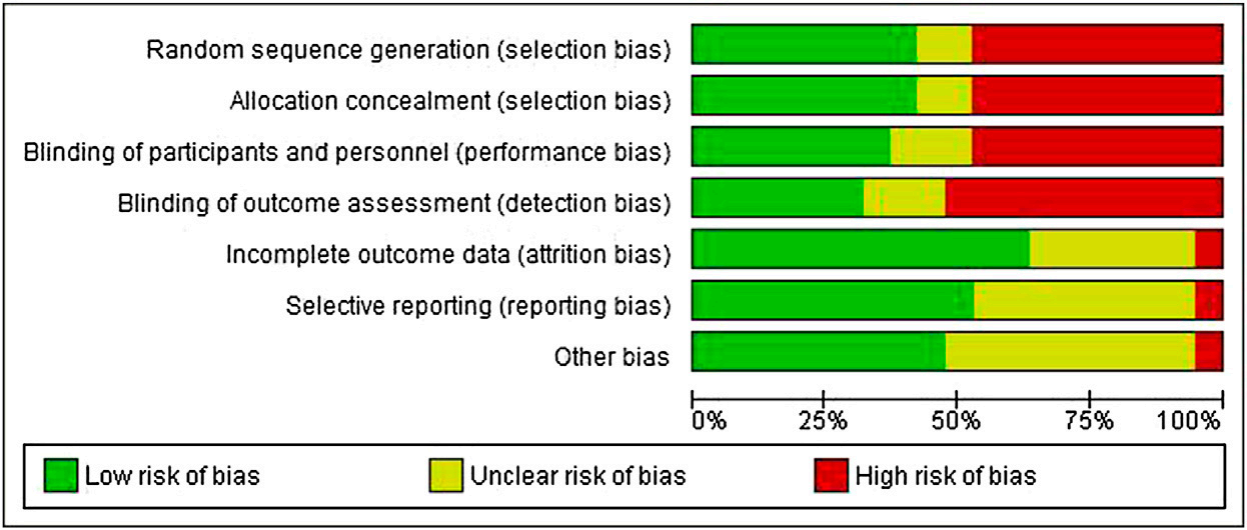
Overall risk of bias.

**FIGURE 3:**
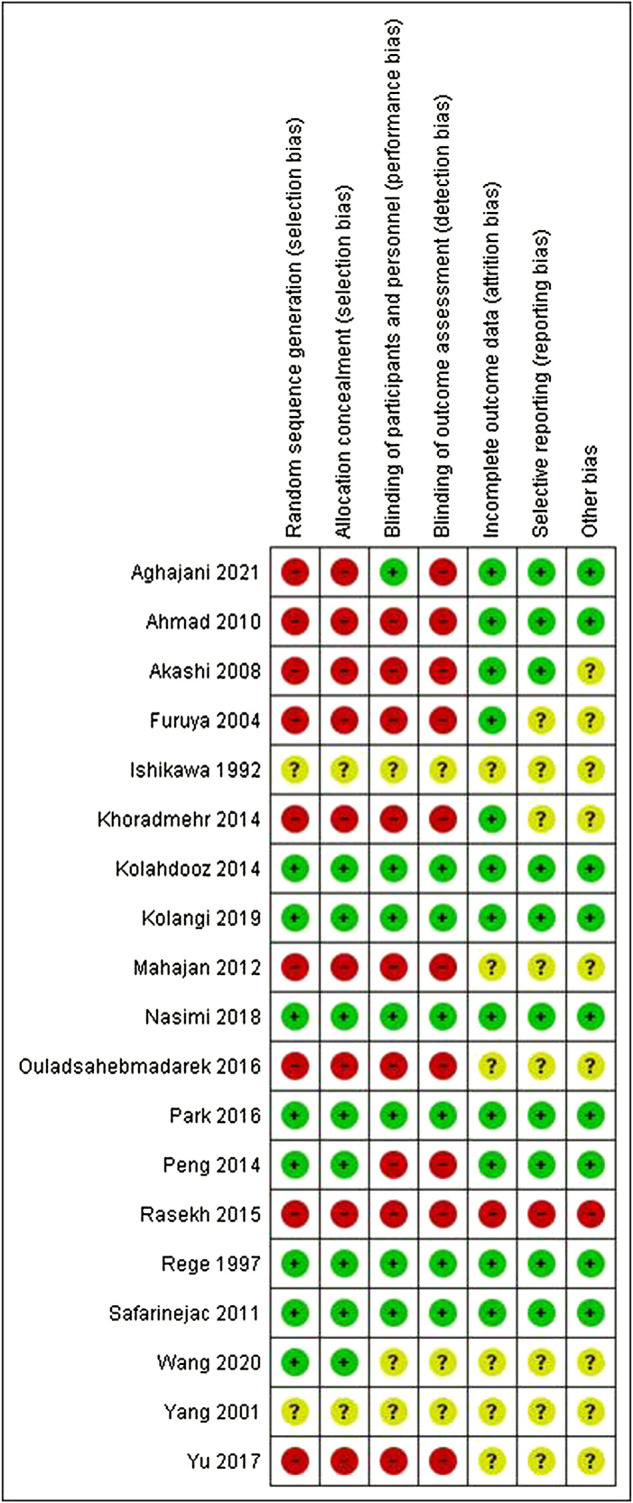
Summary of risk of bias for individual studies.

Figures 2, 3 present ROB for all included RCTs. More than 90% of the studies were categorized as free of attrition bias, reporting bias, and other sources of bias. Performance bias and selection bias were found in only 40% of the studies.

### Effects of herbs on sperm parameters

Following are the details explaining the effects of all the herbs included on sperm parameters. Table 3 summarizes the proposed mechanism of action and common side effects of all the herbs included in this review on improving semen parameters.

**TABLE 3:** Mechanisms of action of herbal interventions to treat male infertility (n = 19).

S. No.	Name of the herb	Place	Study design	Patient	Comparator	Outcome	Proposed MOA	Reference
01	Y-Virilin	India	Double-blind RCT	52	Placebo	Increased sperm concentration	Site of action appears to be the germinal epithelium where Y-virilin promotes spermatogenesis and maturation of sperms	Rege et al. (1997b)
02	Hochu-ekki-to	Japan	Experimental (single-case study)	67	NA	Increased sperm motility and sperm density	Use of Hochu-ekki-to may have effects on decreasing biosynthesis of serum prolactin and estradiol resulting in stimulation of spermatogenesis	Ishikawa et al. (1992)
		Japan	Experimental (single-case study)	22	NA	Increased sperm concentration, sperm motility, and seminal sFas	Increasing sFas leads to reduced apoptosis of spermatozoa, therefore increasing fertility	Furuya et al. (2004)
		Japan	Experimental (single-case study)	20	NA	Increased sperm motility	Use of Hochu-ekki-to may cause a decrease in the cytokine concentration in the body. This decrease in the cytokine concentration leads to improvement in sperm quality, especially sperm motility	Akashi et al. (2008)
03	*Alpinia officinarum*	Iran	Double-blind RCT	76	Placebo	Increased sperm count	Reduces the production of ROS and decreases apoptosis *via* caspase inhibition, upregulation of Bcl‐2, downregulation of Bax expression, cPARP reduction, and inhibition of the ERK and NF‐κB signaling pathways, resulting in increased sperm quality and reduced DNA damage	Kolangi et al. (2019)
04	*Nigella sativa*	Iran	Double-blind RCT	80	Placebo	Increased sperm count, sperm motility, sperm morphology, and semen volume	Neutralizes the ROS in semen and have favorable effects on spermatogenesis	Kolahdooz et al. (2014)
05	*Withania somnifera*	Iran	Triple-blind RCT	100	Treatment group	Increased sperm count, sperm motility, and sperm morphology	It has protective impacts on Leydig cells and spermatogenesis, increases gonad weight, and regulates sexual hormone levels. It enhances alanine transaminase activity, which leads to an increase in the semen alanine content. Alanine protects spermatozoa against oxidative damage and increases sperm count and motility. It stimulates gonadotropin-releasing hormone (GnRH)-producing neurons, increases Leydig cell testosterone production, and improves spermatogenesis	Nasimi Doost Azgomi et al. (2018)
		India	Experimental (single-case study)	150	Control group	Increased sperm concentration, sperm motility, semen volume, and testosterone. Decreased lipid peroxides	Presence of a high concentration of vitamins in the extract generates the ROS scavenging activity. Vitamin A is an antioxidant and works as a detoxifying agent. Vitamin E promotes the defense system against lipoprotein oxidation and helps in improving sperm motility	Ahmad et al. (2010)
06	Aphrodisiac plants	India	Experimental (single-case study)	20	Control group	Increased sperm motility and density and increase in serum FSH, LH, and testosterone	Scavenges free radicals and minimizes damage to sperm cells from ROS.	Mahajan et al. (2012)
07	*Crocus sativus* Linn	Iran	Double-blind RCT	260	Placebo	No improvements	Contains carotenoids that scavenge seminal plasma free radicals by increasing the seminal plasma’s total antioxidant capacity	Safarinejad et al. (2011)
08	TOPALAF	Iran	Experimental (single-case study)	62	Placebo	Increased sperm count, sperm motility, and semen volume	Increases the plasma level of gonadotropins which affect spermatogenesis and inhibit lipid peroxidation and protein oxidation which increases vitamins A, C, and E which produce an antioxidant effect and protect the cells from ROS and enhance semen parameters	Khoradmehr et al. (2014)
09	Gushenyutai	China	RCT	62	Control group	Increased sperm density	It works *via* the Guanyuan (CV 4) acupoint. Acupoint stimulation can dredge meridians, regulate *Qi* and blood, coordinate *Yin* and *Yang*, and promote pathogenic resistance	Peng et al. (2014)
10	Date palm pollen	Iran	Experimental (single-case study)	40	NA	Increased sperm count, sperm motility, and sperm morphology	Presence of nutrients and phytochemicals in DPP stimulates sperm motility and the grading activity of forwarding movement	Rasekh et al. (2015)
11	Qixiong Zhongzi	China	RCT	66	Control group	Increased sperm motility	Significantly reduces spermatogenic cell apoptosis, prevents ROS accumulation, promotes testosterone secretion, and prevents damage to seminiferous tubules	Wang et al. (2020b)
12	Combination of herbs (basil, citrus, onion, *Zingiber*, cinnamon*, Citrullus lanatus*, and *Daucus carota*)	Iran	Experimental (single-case study)	40	NA	Increased sperm count, sperm motility, and sperm morphology	Combination of herbs imparted the antioxidant effect which enhanced spermatogenesis in patients	Ouladsahebmadarek et al. (2016)
13	Tomato juice	Japan	RCT	54	Control	Increased sperm motility, sperm concentration, and semen volume in the tomato group but not in the antioxidant group	Antioxidant effect to improve sperm count and sperm quality	Yu et al. (2017)
14	*Ceratonia siliqua* L. (Carob) syrup	Iran	RCT	60	NA	Increased sperm count, sperm morphology, and sperm motility	Exact MOA is not known but may be due to the antioxidant effect of an increased level of phenolic and flavonoid contents. Improved sperm parameters by increasing the TAC level and reducing the MDA level	Aghajani et al. (2021)
15	Korean red ginseng	South Korea	Double-blind RCT	80	Placebo	Increased sperm concentration, sperm motility, sperm Morphology, and Sperm vitality	Induces spermatogenesis by cAMP-responsive element modulator (CREM) and regulates estrogen and steroid receptors for the proper functioning of estrogen and androgen hormones	Park et al. (2016)
								Lee et al. (2020)
16	Sheng Jing Zhong Zi Tang	China	Experimental (single-case study)	87	NA	Improved conception and sperm quality, and increased testosterone, FSH, and LH	It could dual-directionally regulate the levels of follicle-stimulating hormone (FSH), prolan B luteinizing hormone (LH), testosterone (T), and cortisol (C)	Yang et al. (2001)

### Y-Virilin

The effect of the herbal formulation, Y-virilin, was studied through a double-blind randomized controlled trial in India (Rege et al., 1997a). The study comprised two phases. A total of 52 patients were randomized into two equal groups: a treatment group and a placebo group. The treatment group was given one capsule of Y-virilin twice a day for 6 months. After 6 months, no significant change was observed in sperm motility and serum FSH. An increase in the sperm concentration was detected, and 25% of couples achieved pregnancy.

### Hochu-ekki-to

The effect of Hochu-ekki-to, a traditional Japanese herbal medicine, was reported by three studies conducted in Japan. The first study was conducted through an experimental (single-blind) study design (Ishikawa et al., 1992). An amount of 7.5 g of the herb was administered to 67 patients daily for 3 months. A significant increase in sperm motility and sperm density was noticed. Serum density before and after treatment was 26 ± 32 × 10^6^/ml and 38 ± 38 × 10^6^/ml, respectively. Sperm motility was increased from 43 ± 23 × 10^6^/ml to 54 ± 22 × 10^6^/ml. Serum FSH was not changed.

The second study was also an experimental (single-case) study (Furuya et al., 2004). A total of 22 patients were inducted and treated with 7.5 g of herb daily for 3 months. Sperm count was increased from 28 ± 31 × 10^6^/ml to 42 ± 32 × 10^6^/ml. Also, sperm motility was increased from 32 ± 17% to 39 ± 19%, and a significant increase in seminal plasma soluble Fas (sFas) was observed. No noticeable change was observed in IL-6 and IL-8.

The third study was also an experimental (single-case) study (Akashi et al., 2008). A total of 20 infertile patients were treated with 7.5 g of the herb daily for 3 months. Sperm motility was increased from 33.0 ± 13.5% to 42.6 ± 15.6%, and seminal RANTES, TNF-α, and IL-6 were slightly decreased. All three studies compared the outcome values with the baseline of the same patients.

### Alpinia officinarum

The effect of *A. officinarum*, a Chinese herbal medicine belonging to the Zingiberaceae family, was studied in Iran through a double-blind RCT (Kolangi et al., 2019). This trial comprised 76 males with idiopathic infertility, which was randomized into a treatment group and a placebo group. An amount of 100 mg of the rhizome extract of the herb was administered to the treatment group, while the placebo group received 100 mg of sucrose thrice a day for 12 weeks. No comparable change was observed in sperm motility and semen volume. Sperm count, however, was increased by 62%.

### Nigella sativa

The oil of *N. sativa*, black cumin, belonging to the Ranunculaceae family is believed to have a positive impact on infertility in males. This effect was studied using a double-blind RCT (Kolahdooz et al., 2014). A total of 80 patients were randomized into an intervention group treated with 2.5 ml of oil twice daily, and a placebo group received 2.5 ml liquid paraffin twice daily, respectively, for 2 months. A significant increase in sperm parameters was observed in the treatment group.

### Withania somnifera

An experimental (single-case) study was conducted in India to observe the effect of *Withania somnifera* root powder, a medicinal plant of the Indian subcontinent, with milk (Ahmad et al., 2010). A total of 150 male patients were equally distributed in the intervention group and control group. The intervention group was treated with 5 g herb daily with milk for 3 months. An increase in sperm parameters and hormones from the baseline was observed along with a decrease in lipid peroxides in the patients as compared to their baseline. Another study was conducted in Iran, a triple-blind RCT, to compare the effects of *W*. *somnifera* with pentoxifylline (Nasimi Doost Azgomi et al., 2018). A total of 100 patients were randomized into two treatment groups. Group 1 was treated with six capsules designed in two different colors (containing 5 g of the WS root) in three divided doses; in the pentoxifylline group, the subjects received six capsules in two different colors (containing 800 mg of this drug and placebo), three times a day over 90 days. Then, 12.5% increase in sperm count, 21.42% increase in motility, and 25.56% increase in morphology were observed in group 1, while a lesser increase was noticed in group 2, without affecting the sperm count. The conception rate was 18 and 12% in groups 1 and 2, respectively.

### Aphrodisiac plants

Medicinal aphrodisiac plants, which include *Mucuna pruriens*, *Chlorophytum borivilianum*, and *Eulophia campestris*, were studied through an experimental (single-case) study in India (Mahajan et al., 2012). A total of 20 oligozoospermic patients were equally divided into the treatment group and the control group. Then, 20 mg/kg herbal composition was administered to the treatment group twice daily for 90 days. Sperm density and motility along with serum FSH, LH, and testosterone were significantly elevated after treatment in the patients as compared to their baseline.

### *Crocus sativus* Linn

A double-blind RCT was conducted in Iran to assess the efficacy of saffron (*C. sativus*) (Safarinejad et al., 2011). A total of 260 patients were randomized into an intervention group and a placebo group. An amount of 30 mg of saffron was given to the treatment group twice daily, while 30 mg of starch was given to the placebo group twice a day for 26 weeks. After the treatment period, no comparable improvement was noticed in semen parameters and hormones, therefore showing saffron to be ineffective in treating male infertility.

### Topalaf

Topalaf is a powdered blend of various herbs including *Tribulus terrestris*, *Orchis mascula* root, pollen of *Phoenix dactylifera*, the seed of *Allium ampeloprasum*, and the seed of *Lepidium sativus*, *Amygdalus communis*, and *Ficus carica*. A total of 62 patients were distributed into a treatment group and a placebo group in an experimental study (Khoradmehr et al., 2014). Topalaf was administered to the treatment group thrice a week for 3 months. A total of 12.9% increase in sperm motility and an 8.14% increase in sperm count from the baseline were noticed. However, an insignificant change in sperm morphology was observed. A total of 18.75% of couples became pregnant in the treatment group compared to 3.3% in the control.

### Gushenyutai

Gushenyutai plaster is made from 23 herbs and serves as a transdermal patch. Peng et al. (2014) conducted an RCT in China to assess the extent of the effect of Gushenyutai plaster on male infertility. A total of 62 patients were randomized into a treatment and a control group. The treatment group was given Gushenyutai plaster with comprehensive therapy, and the control group was administered comprehensive therapy for 8 weeks. Significant improvement in sperm density was observed. The pregnancy rate was 38.71 and 16.13%, respectively, in the treatment and control groups.

### Date palm pollen (DPP)

A total of 40 infertile males were inducted into an experimental study conducted by Rasekh et al. (2015). An amount of 120 mg/kg DPP powder capsules were given to patients every other day. After 60 days, a noticeable increase in semen concentration, morphology, and motility was observed in the patients as compared to their baseline parameters. Sperm motility was increased by 4.6%. However, the semen volume did not change significantly.

### Qixiong Zhongzi

The effect of Qixiong decoction, a Chinese medicine, was studied through an RCT in China (Wang et al., 2020b). A total of 66 patients were randomly distributed into a treatment group and a control group. The treatment group was given 150 ml decoction of Qixiong Zhongzi twice a day, while the control group received 1 g of levocarnitine twice a day. No significant change was detected in the semen volume and sperm density after 12 weeks. None of the couples got pregnant. However, a significant increase in sperm motility was observed.

### Korean red ginseng

Park et al. (2016) conducted a double-blinded RCT to assess the efficacy of red ginseng in male infertility. A total of 80 patients were divided into an intervention and a placebo group. The treatment group was given three 500 mg capsules of red ginseng daily for 12 weeks. At the end of the study, all sperm parameters were significantly increased, but no change was found in the plasma hormonal concentrations.

### Compound herbal remedy

Ouladsahebmadarek et al. conducted an experimental study in which 40 patients were given 700 mg capsules of a compound herbal remedy once a day. The compound herbal remedy comprised *Allium cepa, Cinnamomum verum, Zingiber officinale, Ocimum basilicum, Citrullus lanatus*, peel of *Citrus sinensis*, and seeds of *Daucus carota* (Ouladsahebmadarek et al., 2016). A significant increase was observed in sperm parameters of the patients after 6 months, and 17.9% of couples reported pregnancy after treatment.

### Tomato juice

In Japan, an RCT was conducted to evaluate the effect of tomato juice on male infertility (Yu et al., 2017). A total of 54 patients were randomized into two treatment groups and one control group. Treatment group 1 was given 30 mg lycopene (from tomato), and treatment group 2 was given an antioxidant capsule, once daily for 12 weeks. Significant improvement in sperm concentration and motility was detected in treatment group 1 by the sixth week, but no improvement was observed in group 2.

### Ceratonia siliqua

A parallel randomized controlled trial was conducted to determine the efficacy of carob syrup (*C. siliqua*), an evergreen shrub or tree, in comparison with vitamin E as an antioxidant for the treatment of male infertility (Aghajani et al., 2021). This study comprised 60 patients randomized into two treatment groups. Group 1 received 7.5 ml of carob syrup twice a day, while group 2 received 100 mg of vitamin E twice a day for 3 months. The results showed a significant increase in semen parameters and hormonal levels of testosterone along with a significant decrease in malondialdehyde (MDA) in the carob syrup group. The vitamin E group also showed improvement in semen parameters, but it was comparatively less than the carob syrup group. The conception rate was 23% for the carob syrup group versus 13% for the vitamin E group.

### Sheng Jing Zhong Zi Tang

An experimental (single-case) study tested the efficacy of the Chinese decoction, Sheng Jing Zhong Zi Tang, in patients suffering from spermatopathy (Yang et al., 2001). A total of 83 out of 87 (95.4%) spermatopathy patients were treated with the decoction, and 49 out of 87 patients were successful in getting their spouses pregnant. The results also showed that the decoction could have a dual-directional regulatory effect on the levels of follicle-stimulating hormone, luteinizing hormone, testosterone, and cortisol.

## Discussion

According to the extracted data, all of the studies reported an improvement in at least one or more semen quality parameters except one study (Safarinejad et al., 2011) which reported no significant improvement upon administration of saffron. All the studies reported at least one primary outcome (concentration, morphology, or motility) except one study that reported conception and sperm density (Peng et al., 2014). The most number of studies three were conducted on Hochu-ekki-to, followed by two on *Withania somnifera*. Administration of Hochu-ekki-to and *Withania somnifera* resulted in an improvement in sperm motility and concentration and a decrease in cytokines and an improvement in the primary endpoints of semen, respectively. Therefore, these herbs show great promise and should be further studied as a treatment option for male infertility. Table 3 summarizes the proposed mechanism of action in which the herbs included in this review improve semen parameters.

The primary outcomes assessed for infertility include sperm count, motility, and morphology (Buhling et al., 2019). Other possible outcomes include conception, semen volume, hormonal levels (testosterone, follicle-stimulating hormone, luteinizing hormone, etc.), sperm density, vitality, and total motile sperm count (TMSC). There are also various endpoints existing which are indirectly linked to infertility, possibly due to any secondary cause. These include various cytokines and chemokines. For example, seminal RANTES is a chemokine and acts as a chemoattractant for WBCs in various genital infections, in which the excess can lead to infertility (Isobe et al., 2002). On the other hand, TNF-α, which can exhibit genetic polymorphism, leads to decreased sperm motility (NAZ and KAPLAN, 1994; Akashi et al., 2008; Mostafa and Taymour, 2016) whereas IL-6 and IL-8 produced by WBCs are associated with decreased sperm penetration and semen quality, respectively (Eggert-Kruse et al., 2001; Furuya et al., 2004; Akashi et al., 2008). A decrease in sFas can cause an increase in oxidative stress, thus leading to infertility (Furuya et al., 2004; Tawadrous et al., 2013). Collectively, a decreased concentration of aforementioned chemokines except for sFas (it should be increased) may lead to improvement in male infertility.

Three studies (Yu et al., 2017; Nasimi Doost Azgomi et al., 2018; Aghajani et al., 2021) compared the efficacy of herbal intervention with pharmacological (pentoxifylline) or supplement-based (vitamin C, vitamin E, and glutathione) intervention. All three of these studies reported a greater improvement in the herbal intervention group than the pharmacological or supplement-based intervention group. Nonetheless, recently, a systematic review and meta-analysis have been performed which assessed the efficacy of all the pharmacological interventions used in male infertility. According to the review, the combination of clomiphene, zinc, and testosterone, as well as CoQ10, FSH, and tamoxifen yielded the best improvements in male semen parameters (Shahid et al., 2021).

Six studies compared an intervention with a placebo (Rege et al., 1997a; Safarinejad et al., 2011; Khoradmehr et al., 2014; Kolahdooz et al., 2014; Park et al., 2016; Kolangi et al., 2019). All of these studies reported significant improvement in semen parameters, therefore reinforcing the reported efficacy of the herbal interventions used in the study.

Five studies compared an intervention with control (Ahmad et al., 2010; Mahajan et al., 2012; Peng et al., 2014; Yu et al., 2017; Wang et al., 2020b). None of the articles reported any significant improvement in the specified semen endpoints except in one study (Peng et al., 2014) which reported an increase in the sperm density and success of conception in the control group. This improvement in sperm parameters may likely be due to the comprehensive therapy that the control group received. This was the same therapy received by the treatment group with the difference being the addition of the Gushenyutai plaster which contains 23 herbs.

Some of the studies reported side effects (Safarinejad et al., 2011; Park et al., 2016; Kolangi et al., 2019; Wang et al., 2020b). These included common colds, allergic reactions, headaches, hypomania, increased appetite, and nausea. All of these adverse effects were spontaneously resolved and were assumed to not be related to the use of herbal products.

Most of the studies included in this review have shown positive trends in the semen parameters and thus present a strong case of having comparable efficacy and possibly being an alternative to pharmacological interventions in the future. However, it is really difficult to recommend herbal interventions as alternative to pharmacological interventions as it is hard to come to a conclusion about which one is most effective among those. Moreover, there were observed discrepancies with the reported clinical outcomes, and somehow, the results of trials were not comparable because of i) different treatment durations (ranging from weeks to months), ii) monotherapy (single herb vs. combinations) or combined with other therapy, iii) no standard comparative agent, iv) different extracts (no standardization on dose), v) patient number and populations with different extents and severity of infertility, and vi) inconsistent study endpoints. Caution must be exercised, however, in the use of herbs at least until the FDA and WHO approve them.

Further studies are required to compare the efficacy and safety of these herbal drugs to pharmacological drugs as well as to other possible methods (e.g., surgery). All current and future trials should be comprehensive and conform to the CONSORT guidelines (Dworkin et al., 2010). It is suggested that further studies comprise larger sample size, different doses of herbal drugs, and a longer duration of the study to maximally achieve more definitive results regarding the safety and efficacy of these drugs for male infertility.

This review may have been affected by publication bias (inherited from the trials), as is the case for all medical research practices (Easterbrook et al., 1991). Research studies on alternative medicine are more prone to publication bias than other studies (Ernst and Pittler, 1997). A narrative summary is also thought to be susceptible to subjectivity and bias to be limited when the effect size is absent (Graham, 1995). To minimize this possibility, two reviewers discussed the study outcomes, quality indicators, and the effects of shortcomings in the methodology in detail. Any disagreement was resolved by a third reviewer if necessary.

## Conclusion

This systematic review has demonstrated the efficacy of herbal medicines in the treatment of male infertility. Herbs may have a role in the management of male infertility. However, more studies are warranted, and future experimental and clinical studies should be directed toward identifying the specific components and mechanisms by which identified components exert their clinical effects. The preparation of herbal products should be standardized; to ensure a degree of purification of the herbs and thus efficacy, we want to include other kinds of patients or infertility, as in the trial data, and any side effects reported should be well monitored. As far as the current systematic review is concerned, herbal medicines, especially Hochu-ekki-to and *W. somnifera*, are potential candidates for alternative treatment options, subject to further studies on aforementioned parameters.

### Strengths and limitations

Numerous curbs are associated with this study. Due to a lack of resources, non-English studies were not reviewed as it was difficult to translate them to other languages. A combination of data from non-English literature might alter the significance of the current analysis of various male infertility interventions. Lastly, due to diversified types of male infertility along with different herbal interventions, all such interventions were classified into multiple types. Along with all the limitations, our systematic review is the first study establishing a comparison among all available herbal interventions regarding male infertility, and this comprises a very significant aspect of this work.

### Clinical implications

This is perhaps the first study to compare all the available herbal interventions to improve sperm morphology, count, and male infertility health. In addition, this study has also summarized the effects of different herbal interventions which will serve as an ideal approach to optimize the therapy based on the effect size and might be useful in optimizing the cost of therapy as well. This study is of significant value for healthcare providers and policymakers in selecting the perfect blend of herbal interventions for male infertility patients, keeping in view the existing health resources. This review establishes that all herbal interventions had a significantly positive effect on male infertility. There is a need for future experimental studies on these interventions with significant effect sizes so that better pharmacotherapy can be planned to improve the outcome of therapy.

## Data Availability

The original contributions presented in the study are included in the article/Supplementary Material; further inquiries can be directed to the corresponding author.
