# Supplementary material for: A systematic review on the effectiveness of herbal interventions for the treatment of male infertility
Source: Front Physiol. 2022 Nov 4;13:930676. doi: 10.3389/fphys.2022.930676 (PMC9672875; doi:10.3389/fphys.2022.930676)
Supplement: Supplementary file 1 [file Table1.DOCX]

**A Systematic Review on the effectiveness of Herbal Interventions for the treatment of Male Infertility**

**Mr. Muhammad Nabeel Shahid* (Corresponding author and first author)**

Affiliations:

1. Department of Pharmacy Practice, Faculty of Pharmacy, Universiti Teknologi MARA (UiTM), Cawangan Selangor, Puncak Alam Campus, 42300 Bandar Puncak Alam, Selangor, Malaysia.

2. Department of Pharmacy Practice, Institute of Pharmaceutical Sciences, University of Veterinary and Animal Sciences, 54000, Lahore, Punjab, Pakistan.

Email address:

nabeelshahidk@hotmail.com

**Hassaan Shahzad Afzal**

Affiliations:

1. Department of Pharmacy Practice, Institute of Pharmaceutical Sciences, University of Veterinary and Animal Sciences, 54000, Lahore, Punjab, Pakistan.

Email address: hsahb1398@gmail.com

**Bareerah Farooq**

Affiliations:

1. Department of Pharmacy Practice, Institute of Pharmaceutical Sciences, University of Veterinary and Animal Sciences, 54000, Lahore, Punjab, Pakistan.

Email address: bareerahfarooq2020@gmail.com

**Muhammad Rehan Yousaf**

Affiliations:

1. Department of Pharmacy Practice, Institute of Pharmaceutical Sciences, University of Veterinary and Animal Sciences, 54000, Lahore, Punjab, Pakistan.

Email address: rehanyousaf668@gmail.com

**Muhammad Rauf Ijaz**

Affiliations:

1. Department of Pharmacy Practice, Institute of Pharmaceutical Sciences, University of Veterinary and Animal Sciences, 54000, Lahore, Punjab, Pakistan.

Email address: raoofijaz123@gmail.com

**Talha Ali Shafqat**

Affiliations:

1. Department of Pharmacy Practice, Institute of Pharmaceutical Sciences, University of Veterinary and Animal Sciences, 54000, Lahore, Punjab, Pakistan.

Email address: talhaalishafqat@gmail.com

**Dr. Tahir Mahmood Khan (Author)**

Affiliations:

1. Department of Pharmacy Practice, Institute of Pharmaceutical Sciences, University of Veterinary and Animal Sciences, 54000, Lahore, Punjab, Pakistan.

2. School of Pharmacy, Monash University, Subang Jaya, Malaysia

Email address:

tahir.khan@uvas.edu.pk

**Dr. Chin Fen Neoh (Author)**

Affiliations:

1. Department of Pharmacy Practice, Faculty of Pharmacy, Universiti Teknologi MARA (UiTM), Cawangan Selangor, Puncak Alam Campus, 42300 Bandar Puncak Alam, Selangor, Malaysia.

Email address:

chinfenneoh@gmail.com

**Dr. Qi Ying LEAN (Author)**

Affiliations:

1. Faculty of Pharmacy, Universiti Teknologi MARA (UiTM), Cawangan Pulau Pinang, Kampus Bertam, Malaysia.

2. Vector-Borne Diseases Research Group (VERDI), Pharmaceutical and Life Sciences CoRe, Universiti Teknologi MARA (UiTM), Shah Alam, Malaysia.

Email address:

leanqiying@uitm.edu.my

**Dr. Allah Bukhsh Malik (Author)**

Affiliations:

1. Department of Pharmacy Practice, Institute of Pharmaceutical Sciences, University of Veterinary and Animal Sciences, 54000, Lahore, Punjab, Pakistan.

2. School of Pharmacy, Monash University, Subang Jaya, Malaysia

Email address:

abukhsh@uvas.edu.pk

**Dr. Mahmathi Karuppannan (Author)**

Affiliations:

1. Department of Pharmacy Practice, Faculty of Pharmacy, Universiti Teknologi MARA (UiTM), Cawangan Selangor, Puncak Alam Campus, 42300 Bandar Puncak Alam, Selangor, Malaysia.

Email address:

mahmathi@uitm.edu.my

Supplementary material: Contents

Appendix 1 Search strategy

**APPENDIX I: SEARCH STRATEGY**

**Search Term**

| Search Items | | Search Engines | | | | | | |
| --- | --- | --- | --- | --- | --- | --- | --- | --- |
|  |  | **PubMed** | **Scopus** | **Cochrane Library** | **Embase** | **EBSCOhost** | **Ovid Medline** | **Google Scholar** |
| ("infertility"[MeSH Terms] OR "infertility"[All Fields]) AND ("oligospermia"[MeSH Terms] OR "oligospermia"[All Fields]) AND "humans"[MeSH Terms] | Result | 5535 | 8200 | 207 | 4733 | 3049 | 4777 | 15 |
|  | Chosen | 5499 | 4786 | 69 | 1810 | 403 | 4301 | 11 |
| ("infertility"[MeSH Terms] OR "infertility"[All Fields]) AND ("azoospermia"[MeSH Terms] OR "azoospermia"[All Fields] OR "azospermia"[All Fields]) AND "humans"[MeSH Terms] | Result | 4709 | 352 | 18 | 6523 | 3705 | 8351 | 80 |
|  | Chosen | 2352 | 173 | 14 | 2212 | 641 | 4375 | 25 |
| ("infertility"[MeSH Terms] OR "infertility"[All Fields]) AND ("oligospermia"[MeSH Terms] OR "oligospermia"[All Fields] OR "oligozoospermia"[All Fields]) AND "humans"[MeSH Terms] | Result | 5992 | 5288 | 61 | 1494 | 1137 | 2672 | 15 |
|  | Chosen | 236 | 2316 | 10 | 115 | 51 | 424 | 10 |
| ("infertility"[MeSH Terms] OR "infertility"[All Fields]) AND ("oligospermia"[MeSH Terms] OR "oligospermia"[All Fields] OR "oligoasthenoteratozoospermia"[All Fields]) AND "humans"[MeSH Terms] | Result | 5677 | 1241 | 38 | 434 | 271 | 707 | 6 |
|  | Chosen | 99 | 108 | 14 | 82 | 20 | 206 | 4 |
| ("infertility"[MeSH Terms] OR "infertility"[All Fields]) AND (("genitalia"[MeSH Terms] OR "genitalia"[All Fields] OR "genital"[All Fields]) AND ("disease"[MeSH Terms] OR "disease"[All Fields])) AND "humans"[MeSH Terms] | Result | 3312 | 20352 | 108 | 22 | 1631 | 74 | 2 |
|  | Chosen | 2859 | 16408 | 59 | 7 | 383 | 44 | 1 |
| ("infertility"[MeSH Terms] OR "infertility"[All Fields]) AND ("semen"[MeSH Terms] OR "semen"[All Fields]) AND "humans"[MeSH Terms] | Result | 10225 | 41244 | 876 | 13428 | 11509 | 21294 | 373 |
|  | Chosen | 7123 | 22701 | 322 | 4288 | 1307 | 12591 | 257 |
| ("infertility"[MeSH Terms] OR "infertility"[All Fields]) AND ("oligospermia"[MeSH Terms] OR "oligospermia"[All Fields] OR ("low"[All Fields] AND "sperm"[All Fields] AND "count"[All Fields]) OR "low sperm count"[All Fields]) AND "humans"[MeSH Terms] | Result | 6036 | 7319 | 79 | 143 | 239 | 483 | 3 |
|  | Chosen | 161 | 4161 | 20 | 33 | 1 | 181 | 2 |
| ("infertility"[MeSH Terms] OR "infertility"[All Fields] OR "subfertility"[All Fields]) AND ("oligospermia"[MeSH Terms] OR "oligospermia"[All Fields]) AND "humans"[MeSH Terms] | Result | 5521 | 1224 | 29 | 318 | 135 | 402 | 0 |
|  | Chosen | 0 | 26 | 2 | 28 | 1 | 23 | 0 |
| ("infertility"[MeSH Terms] OR "infertility"[All Fields] OR "subfertility"[All Fields]) AND ("azoospermia"[MeSH Terms] OR "azoospermia"[All Fields]) AND "humans"[MeSH Terms] | Result | 4692 | 9 | 0 | 290 | 86 | 694 | 1 |
|  | Chosen | 23 | 0 | 0 | 29 | 0 | 16 | 1 |
| ("infertility"[MeSH Terms] OR "infertility"[All Fields] OR "subfertility"[All Fields]) AND ("oligospermia"[MeSH Terms] OR "oligospermia"[All Fields] OR "oligozoospermia"[All Fields]) AND "humans"[MeSH Terms] | Result | 5993 | 1119 | 29 | 93 | 52 | 333 | 3 |
|  | Chosen | 1 | 7 | 0 | 4 | 3 | 2 | 2 |
| ("infertility"[MeSH Terms] OR "infertility"[All Fields] OR "subfertility"[All Fields]) AND ("oligospermia"[MeSH Terms] OR "oligospermia"[All Fields] OR "oligoasthenoteratozoospermia"[All Fields]) AND "humans"[MeSH Terms] | Result | 5677 | 317 | 1 | 30 | 17 | 95 | 0 |
|  | Chosen | 0 | 2 | 0 | 4 | 0 | 0 | 0 |
| ("infertility"[MeSH Terms] OR "infertility"[All Fields] OR "subfertility"[All Fields]) AND (("genitalia"[MeSH Terms] OR "genitalia"[All Fields] OR "genital"[All Fields]) AND ("disease"[MeSH Terms] OR "disease"[All Fields])) | Result | 4162 | 1942 | 15 | 2 | 38 | 10 | 0 |
|  | Chosen | 780 | 273 | 2 | 1 | 3 | 1 | 0 |
| ("infertility"[MeSH Terms] OR "infertility"[All Fields] OR "subfertility"[All Fields]) AND ("semen"[MeSH Terms] OR "semen"[All Fields]) | Result | 11781 | 6031 | 119 | 1110 | 508 | 2163 | 12 |
|  | Chosen | 1552 | 463 | 19 | 199 | 17 | 218 | 9 |
| ("infertility"[MeSH Terms] OR "infertility"[All Fields] OR "subfertility"[All Fields]) AND ("oligospermia"[MeSH Terms] OR "oligospermia"[All Fields] OR ("low"[All Fields] AND "sperm"[All Fields] AND "count"[All Fields]) OR "low sperm count"[All Fields]) | Result | 6508 | 1465 | 16 | 9 | 5 | 76 | 0 |
|  | Chosen | 321 | 76 | 3 | 3 | 0 | 0 | 0 |
| Subfertile[All Fields] AND ("oligospermia"[MeSH Terms] OR "oligospermia"[All Fields]) | Result | 167 | 1449 | 29 | 242 | 169 | 377 | 0 |
|  | Chosen |  | 49 | 0 | 20 | 4 | 59 | 0 |
| Subfertile[All Fields] AND ("azoospermia"[MeSH Terms] OR "azoospermia"[All Fields] OR "azospermia"[All Fields]) | Result | 107 | 90 | 0 | 197 | 112 | 653 | 0 |
|  | Chosen | 10 | 0 | 0 | 19 | 1 | 57 | 0 |
| Subfertile[All Fields] AND ("oligospermia"[MeSH Terms] OR "oligospermia"[All Fields] OR "oligozoospermia"[All Fields]) | Result | 189 | 1364 | 11 | 85 | 67 | 345 | 0 |
|  | Chosen | 1 | 23 | 0 | 4 | 0 | 10 | 0 |
| Subfertile[All Fields] AND ("oligospermia"[MeSH Terms] OR "oligospermia"[All Fields] OR "oligoasthenoteratozoospermia"[All Fields]) | Result | 177 | 302 | 1 | 21 | 16 | 106 | 3 |
|  | Chosen | 0 | 2 | 0 | 3 | 0 | 3 | 2 |
| Subfertile[All Fields] AND (("genitalia"[MeSH Terms] OR "genitalia"[All Fields] OR "genital"[All Fields]) AND ("disease"[MeSH Terms] OR "disease"[All Fields])) | Result | 65 | 1690 | 15 | 0 | 27 | 6 | 0 |
|  | Chosen | 7 | 268 | 0 | 0 | 0 | 1 | 0 |
| Subfertile[All Fields] AND ("semen"[MeSH Terms] OR "semen"[All Fields]) | Result | 747 | 6861 | 119 | 5078 | 781 | 2229 | 82 |
|  | Chosen | 131 | 872 | 1 | 120 | 28 | 346 | 60 |
| Subfertile[All Fields] AND ("oligospermia"[MeSH Terms] OR "oligospermia"[All Fields] OR ("low"[All Fields] AND "sperm"[All Fields] AND "count"[All Fields]) OR "low sperm count"[All Fields]) | Result | 224 | 1339 | 17 | 11 | 16 | 76 | 0 |
|  | Chosen | 0 | 78 | 0 | 0 | 1 | 2 | 0 |

**Supplementary Table 1: Reasons for study exclusion after full-text assessment (n=607)**

| **Database** | **Number of records** |
| --- | --- |
| Prescreening record | **72** |
| Irrelevent articles (conference papers/proceedings, letter to the editor and study protocols) | **12** |
| Article excluded on the basis of eligibility assessment | **40** |
